# The social well-being of nurses shows a thirst for a holistic support: A qualitative study

**DOI:** 10.3402/qhw.v10.27749

**Published:** 2015-09-15

**Authors:** Naser Mozaffari, Hamid Peyrovi, Nahid Dehghan Nayeri

**Affiliations:** 1 Department of Medical-Surgical Nursing, School of Nursing and Midwifery, Tehran University of Medical Sciences, Tehran, Iran; 2 Nursing Care Research Center, Department of Critical Care Nursing, School of Nursing and Midwifery, Iran University of Medical Sciences, Tehran, Iran; 3 Department of Nursing Management, Nursing and Midwifery Care Research Center, Tehran University of Medical Sciences, Tehran, Iran

**Keywords:** Social well-being, support, nurses, qualitative study

## Abstract

**Introduction:**

Social well-being is one of the important aspects of health. In fact, this is a reflection of experience in a social environment, indicating how social challenges are determined. In other words, social well-being is an explanation of people's perception and experience of being in a good situation, satisfaction with the structure, and social interaction. This qualitative study intended to explore nurses’ experience of social well-being.

**Methods:**

Qualitative content analysis was used to conduct the study. Through purposive sampling, a total of 18 nurses with various clinical experiences participated in semi-structured interviews. The data were analysed using the five-step, qualitative content analysis introduced by Graneheim and Lundman.

**Results:**

The main theme extracted from the data analysis was “thirst for a holistic support” in nurses. It consisted of two subthemes including internal support (family's support, colleague's support, and organizational support) and external support (society's support and media's support).

**Conclusions and discussion:**

Nurses’ experiences in shaping their social well-being show that nurses need support in order to rebuild their social well-being. It is supported in partnership with the media, the community, health-related organizations, and by nurses and family. This improves job satisfaction, hope, motivation, commitment, and confidence so as to ultimately facilitate improvement of social well-being of nurses.

Healthy people play a central role in social development. Human beings are affected by many factors which can be analysed and investigated better in social areas rather than in medical areas. The social aspect of health is a significant factor requiring more serious attention than all other aspects of the health (Mozaffari, Dadkhah, Shamshiri, Mohammadi, & Nayeri, 2014; Szreter & Woolcock, 2004).

In fact, this is a reflection of people's experience in the social environment, and it indicates how social challenges are resolved (Keyes, 1998). In other words, social well-being is a detailed description of people's perception and experience of being in a good condition, satisfaction with the structure, social interaction, and feeling of happiness (Law, Steinwender, & Leclair, 1998). Such description is influenced by social, cultural, political, and historical contexts (Prudant, 2012). Despite a history of more than one century, nursing has not yet found its deserved position, for which it needs particular support.

Rahimaghaee, Nayeri, and Mohammadi (2010) considered managers and community support necessary in order to develop and promote the position of the nursing profession (Rahimaghaee et al., 2010). To achieve such a goal, human resource managers are required to offer necessary support for nursing as the largest profession in the medical and healthcare system (Oshvandi et al., 2008; Sodeify, Vanaki, & Mohammadi, 2013) in order to provide efficiency and effectiveness for healthcare systems as one of the major concerns in many countries (Lu, While, & Louise Barriball, 2005).

In a study on the rate of social support for nurses, Rezaee and Ghajeh (2009) found that 10.7% had good support. Nurses expect more support from the society, their organization, and their colleagues, based on the importance and type of their duty. It has been shown that nurses actually consider such support among the factors potentially reducing their job stress and increasing their satisfaction and health (Rezaee & Ghajeh, 2009).

Furthermore, Varaei, Vaismoradi, Jasper, and Faghihzadeh (2012) reported a dissatisfaction rate of 77.6% among nurses regarding their social prestige and 76.6% regarding lack of respect from the society. In addition, 62.6% of these nurses thought about leaving their jobs despite their positive attitude toward nursing (Varaei et al., 2012). Such low level of support is not only considered a threat to social status and health of the nurses but also indirectly affects how they carry out their treatment tasks (Jannati, Mohammadi, & Seyedfatemi, 2011; Oshvandi et al., 2008).

Sodeify et al. pointed out that the main factors inhibiting supporting efforts were inappropriate organizational climate, low dignity, poor work conditions, and managers’ unawareness of individual and professional values (Sodeify et al., 2013). Considering the previously mentioned issues, the social interactions, general satisfaction, happiness, and other factors of social health, this aspect of health will be reinforced in the shade of support. In this regard, the current study aimed to explore the experiences of social well-being among nurses.

## Materials and methods

This study was conducted as part of a nursing PhD dissertation at Tehran University of Medical Sciences (TUMS), Iran. A qualitative content analysis was used to conduct the study. The methodological approach was content analysis described by Graneheim and Lundman (2004).

We framed the research question based on the aim of the study. The central question of the study was “How is your perception and experience of social well-being as a nurse?” And “How would you describe your social interactions?”

### Study participants

Using a purposive sampling method, a total of 18 nurses with clinical work experience were selected. The mean and the standard deviation of participants’ age and work experience were 38.83 (±7) years and 15.94 (±6.38) years, respectively. The interviews were carried out from April 2013 to September 2014. Characteristics of the participants have been presented in Table I.

**Table I:** Characteristics of the participants.

Participant	Gender	Age	Work experience	Degree
1	Male	50	28	BS
2	Male	39	13	BS
3	Female	42	18	BS, MSc.
4	Female	41	19	BS, MSc.
5	Male	37	15	BS
6	Female	46	20	BS
7	Female	30	12	BS
8	Female	31	10	BS
9	Female	33	11	BS
10	Male	37	13	BS
11	Male	40	16	BS
12	Male	39	14	BS, MSc.
13	Male	45	21	BS
14	Female	41	18	BS, MSc.
15	Female	45	23	BS
16	Male	48	25	BS
17	Male	23	1	BS
18	Female	32	10	BS, MSc., PhD

### Data collection

Data were collected through interviews, which were conducted until a data saturation point was achieved. The participants were selected with the maximum various sampling in terms of work experience, age, sex, education, and so on. The interviews were semi-structured (Speziale, Streubert, & Carpenter, 2011). This type of interview is appropriate for qualitative studies because of its flexibility and depth. Semi-structured interviews provide an opportunity for participants to fully explain their experience about the phenomenon under study. All the interviews were conducted by the main researcher. It first started with a general, open-ended question “How is your perception and experience of social well-being as a nurse?” The next follow-up questions focused on further clarification on the main research question, based on the information provided by the participants. Duration of interviews varied between 45 and 75 min based on the interview conditions. The interviews took place face-to-face with prior consent of the participants in the designated place. The responses were then analysed word-by-word using MAXQDA10, a text analysis software application. The software has four parts to manage data, including Document System, Code System, Document Browser, and Retrieve System. The audiotaped interviews were typed in the document section. They were immediately assessed so as to find the meaning of units/codes based on the method proposed by Graneheim and Lundman (2004). This task required an in-depth immersion in the qualitative data by the researchers (Denise, Beck, & Hungler, 2001).

### Data analysis

In this study, the data were collected and analysed simultaneously through qualitative content analysis using the five-step method proposed by Graneheim and Lundman (2004). In the first step, the recorded interviews were immediately transcribed word-by-word, and they were used as the main data of the research. In the second step, the recorded voices were listened several times, handwritten texts were frequently reviewed, and a decision was made to divide the text into meaningful units. In the third step, the meaningful units were abstracted and they were coded. Considering the participants’ experiences, explicit and implicit concepts were specified in the form of sentences or paragraphs from the words and signifier codes. Then, the outputs were coded and summarized. In the fourth step, the codes were classified into subthemes based on comparisons regarding their similarities and differences. In the final step, themes were formulated as the expression of the latent content of the text (Graneheim & Lundman, 2004).

This study involved prolonged involvement, various methods of data collection. (i.e., notes in the field and recorded interviews), review by the observer, and constant comparative analysis of the data so as to ensure the credibility of data. A member check was performed using additional comments of the colleagues and reviewing handwritten texts by participants. The conformability of the findings was determined by submitting the reports, handwritten texts, and notes to two nursing faculty members. The research transferability was confirmed by an in-depth description of data.

Furthermore, the accuracy of the survey, data collection, and analysis based on the systematic methodology of research were not only confirmed by the research supervisor, but were also approved by several experts in qualitative researches at face-to-face, semi-annual meetings.

### Ethical consideration

This study was approved by the ethics committee at Tehran University of Medical Sciences (Project Number: 91/130/3171). The consent of the related authorities was obtained before starting the research. At the beginning of an interview, each participant was informed about the research purpose and method of interview. Moreover, the participants were ensured about the data confidentiality and their right to be included in or excluded from the study. The participants expressed their informed consent and signed a consent form. The interview times were arranged in a way to not interrupt the participants’ daily plan. Finally, the identities were protected by using code names.

## Results

The data analysis showed that support was like an umbrella covering different levels of the nurses’ social well-being. Based on the participants’ experiences, thirst for a holistic support includes different classes (Table II).

**Table II:** Categories leading to nurse's social well-being.

Categories	Subcategories	Theme
Internal support	Family's support	Thirst for a holistic support
	Colleague's support	
	Organizational support	
External support	Public support	
	Media's support	

Most of the participants stated that their feeling of social well-being would improve by receiving internal support (i.e., protective factors related to the individual and the organization) including family, colleagues, and organization support as well as external support (i.e., supportive factors outside the individual and the organizational levels) including public and the media support. Moreover, the holistic support for nurses can be realized through creation of satisfaction and hope, confidence, motivation, and pleasure so as to provide social well-being.

### Internal support

#### Family support

Among the effective resources in the nurses’ social well-being, the significance of family support was clearly expressed in the interviews. The nurses receiving respect and support from the family and job had greater feelings of acceptance and satisfaction.

The nurses’ family members including father, mother, sisters, and brothers for single individuals as well as spouse for married ones, helped the nurse to select and continue his/her field of study. The sympathy and companionship of the family members toward the nurses when facing problems, such as giving good spirits, creating a comfortable environment to relax after rotation, and supporting when they are hopeless will increase tolerance and satisfaction among the nurses. In this regard, participant 4 stated:

As a small society, my family supported me. Maybe if they didn't do so, I couldn't have succeeded. When my relatives especially my family encourage me and provide me home comfort, I regain the power to continue somehow and express myself.

Another participant promoted from an assistant nurse to nurse considers her husband the most important factor for her success. She said;

I did continue my study from assistant nursing to nursing as I was supported by my husband, especially when my child was sick and I couldn't leave the hospital. He got a leave from his work in order to provide me with the opportunity to continue my work and study with peace of mind. (Participant 6)

Family's support and attitude toward the nursing profession increased motivation and self-confidence in the participants and provided them with an appropriate ground to continue their job. Participant 9 argued this as follows:

My family totally supported me to select nursing as my field of study. My father described nursing for me in such a way that I was insisting that I am definitely going to choose nursing and nothing else.

However, one of the participants considered intensive night shifts in nursing among disturbing factors in his family relationships, stating:

Intensive shifts and night works in nursing are really exhausting and cause physical and mental fatigue, interrupting social and family relationships. These issues are problematic without family's support. (Participant 5)

#### Colleague support

Colleagues’ support played an important role in improving the social well-being of the participants. This approach provided the grounds for the nurse to build effective and pleasant relationships and attendance by bringing hope and increasing self-confidence. Participant 12 stated:

As the representative of nurses, I frequently experienced different conditions where I needed support from my colleagues to make decisions. For example, a manager had misbehaved a nurse in a hospital and I asked my colleagues to protest together so as to support that nurse. This happened through a support from majority of nurses. It created self-confidence and encouragement to follow up their later demands. (Participant 12)

The support by colleagues and establishment of a communicative network to help each other is one of the methods used by nurses to influence the society and demonstrate their capability. Participant 8 states:

I am acknowledging myself as a nurse with satisfaction, because whenever I need to do something for a client, it would happen easily by calling my colleagues and requesting them to help my client in the hospital. Their respectful behavior with the client I introduced to gives them the same dignity and respect for me and my colleagues, which is really encouraging.

Furthermore, mutual supporting would encourage the nurses while creating self-confidence, as Participant 7 stated:

When a nurse can rely on a strong support, he/she feels power. In the hospital where I work, the supervisor really supports the nurses. My colleagues trust highly in each other; this type of support could give self-confidence to anybody. (Participant 7)

#### Organizational support

Organizational support created a positive impression and satisfaction in the participants, leaving a substantial effect on the acceptance and the desire to continue nursing. Participant 9 stated:

Of course, the support from organization and working environment has a positive effect on me; I will feel fatigue or vitality depending on the amount of support I receive after finishing my work. For example, I had a problem resolved by the hospital chief, it made me fascinated in my job and committed to continue my career at this medical center. (Participant 9)

Understanding and caring about work conditions by the managers is one of the supporting approaches adopted by the organization. Participant 6 stated:

The managers of the hospital frequently visited my ward without any prior notice and saw that I am working with minimum staff and maximum energy; they always appreciated me and I continued my efforts with motivation and enthusiasm as a nurse. (Participant 6)

Moreover, the support can foster motivation and satisfaction in the nurses. Participant 2 expressed that as:

I myself got motivated totally through even a little support I received. For example, an appreciation by a manager has made me more satisfied, so I tried to provide others with a better service. (Participant 2)

Despite the positive role of support in most of the participants, poor organizational support as well as discrimination can spread frustration among the nurses. They expected the system to treat its staff fairly and to plan appropriate supporting rules. One of the participants stated this as follows:

The healthcare system in Iran has frustrated the nurses’ community due to inadequate support, injustice and discrimination in moral and material valuation. It is as if there is a feudalism system ruling and everything is decided by the physicians; they are looking to other segments of the medical staff as second level employees. (Participant 5)

### External support

The external support as a separate category consists of two dimensions including society's support and media's support. Nurses consider the support provided by the society and the media in addition to intra-organizational and family support as an effective factor contributing to their social well-being. They express such a fact in their accounts of professional experience.

#### Society's support

One of the factors contributing to social well-being involves the society's support in accepting, respecting, and valuing an individual within a professional group. As health service providers, the nurses expect to be considered valuable individuals.

Now and after a decade, I'm more satisfied due to understanding the nursing and good support from people besides mutual respect. For example, when a patient appreciates and prays for me, I find out that there is someone looking at my work and counting on me; which brings me so much hope. (Participant 8)

Life experiences of the participants showed that they earn almost all of the professional prestige from the perspectives of the community. For example, Participant 14 said:

Prestige and social support are very important to be seen. Physiological requirements are somehow important but the next significant issue is the social prestige and value since it represents how people living around us think. (Participant 14)

However, some participants showed different and unreal behavior in introducing themselves as a nurse due to their fear of not being supported and/or common misconceptions about nursing. Participant 16 declared:

If I were in a place where I got respected as a human and not as a person doing a certain job, I would introduce myself comfortably. But if the people want to know what my job is, it depends on the person who asks such a question. I mean if the questioner is someone who knows nothing about nursing, I will say that I am a physician. However, if such a person is an informed one, I will introduce myself as a nurse. (Participant 18)

Whenever I find myself in a crowd of people, I wouldn't say I'm a nurse, because I think people would look at me negatively and do not provide me with a particular support and respect that I expect. (Participant 16)

### Media's support

Nowadays, the media, and in particular TV programs constitute an undeniable part of people's life. The findings of this study showed that the nurses experienced many positive and negative forms of effects from the media for being a nurse. Participant 9 stated:

The TV series ‘Nurses’ and the films showing scientific nursing have to some extent increased the people's understanding of nursing. I can feel this in their expectations from nursing. Many of such works should be done in the media in order to introduce nursing to people and make them respected and trusted by the society. (Participant 9)

The media encourage a new belief and attitude in the society. So long as such beliefs are positive, they have a desired effect; otherwise, they create a negative attitude in the society. One of the participants argued in this respect:

When the movie ‘Shokaran’ was screened as a negative story of a female nurse, some of my colleagues didn't attend their working shifts as a sign of objection. Their husbands called me as the supervisor and told me angrily that this movie has created a bad image and mentality of the nurses, our wives wouldn't like to continue their work as nurses. (Participant 1)

As an effective system in the people's behavior, thoughts, beliefs, and public culture, the media should direct the society toward valuation and trusting in the individuals and professions. Another participant said:

The major problem of nursing is the attitude of the society toward nurses, which has been created by the media. In most cases, the media provides an inappropriate and non-scientific view of nursing and even depict the baby-sitter and housemaid as a nurse. (Participant 11)

Such representation by the media makes people mistrust nursing knowledge. In this respect, a participant stated:

Because of media, people have lost their trust in the nurses’ knowledge by providing a non-scientific image of the nurses. Whenever the hospital visitors ask a nurse about a patient, they are referred to the physician for the answer. (Participant 17)

## Discussion

The results of this study showed that the nurses’ social well-being is closely related to individual, organizational, social, and media factors. The support provided by such factors can help nurses to feel social well-being in the form of satisfaction, motivation, self-confidence, self-esteem, hope, and success within individual and social interactions. These findings were confirmed by those obtained in other studies. Rigby considered the mental and social support very effective in the people's health (Rigby, 2000). This support will not only maintain health among nurses but will also lead to professional development (Hannes et al., 2007). The participants believed that any support for nurses will affect their health and in particular their social health, thus strengthening their satisfaction level. The results of the study conducted by Makros showed that such support plays an essential role in nurses’ feeling of healthiness especially in its social aspect (Makros & McCabe, 2001). The participants asserted that family's support, besides other supporting aspects, brought about hope, success, and acceptance. In fact, they would not be able to continue their activity if such support did not exist. In their investigations, Torres and Solberg (2001) showed that the family's support led to higher self-efficacy, social cohesion, stress reduction, and health promotion among participants (Torres & Solberg, 2001; Valizadeh, Dadkhah, Mohammadi, & Hassankhani, 2014). In a study carried out by Berkman, social and family support and strong relationships were reported to be significant sources of individual health (Berkman, 1995).

In addition to family support, the participants indicated that support provided by colleagues is effective in their health as well as the quality of their work. An investigation by Voorhees et al. (2005) considered the supportive and communicative networks developed between colleagues as an effective factor on the individuals’ activity and health (Voorhees et al., 2005). Furthermore, support from friends and colleagues for the individuals and groups who pay little attention to themselves will provide a good understanding of the problem, thus increasing their capability to make a change (Mead, Hilton, & Curtis, 2001). This can be very helpful for nurses and may strengthen their social well-being by increasing change capacity and social relations. In their study, Repper and Carter (2011) concluded that support from a colleague will help the nurses achieve numerous developments in different life aspects.

The third aspect of internal support involves the organization. The participants consider the support provided by their corresponding organization as a highly essential factor. It indicated that such support affected the nurses in different aspects, bringing to them greater satisfaction, motivation, and trust. The results achieved by Arons et al. suggested that the supporter's organizational culture is one of the underlying factors contributing to innovation and employee acceptance (Aarons & Sawitzky, 2006). Moreover, Oshvandi et al. reported that poor support is one of the main reasons for lack of career incentive (Oshvandi et al., 2008). In their study on 1363 samples, Burke and Greenglass (2001) showed that the nurses enjoying higher support from the hospital enjoyed greater satisfaction, job security, and lower level of psychological burnout. Furthermore, Laschinger and Havens (1997) considered health care of nurses provided by the organization as a factor contributing to higher efficiency and achievement of ideal organizational results, including commitment and satisfaction. It was emphasized that managers ought to care about the health of their staff in order to improve their professional conditions and effectiveness (Laschinger & Havens, 1997).

Participants of this study suggested that inequality at the workplace and difference between incomes are signs of poor organizational support, this in turn affects their performance and health status, leading to a feeling of frustration. The results of this study are consistent with those of other investigations. Elovainio, Kivimäki, and Vahtera (2002) argued in their study that as the fairness perceived by the employees increases, there was lower absenteeism rate and chance of dangerous behavior as compared to those feeling discrimination (Elovainio et al., 2002). Unfairness will lead to conflict in how individuals carry out their tasks, thus endangering their health. On the other hand, organizational support will pave the way for personal and external success of the individual while it leaves a positive impact on the organization (Grant-Vallone & Ensher, 2001). The researchers have also confirmed the positive relationship between the support, health, and organizational commitment. Panaccio and Vandenberghe (2009) reported in a longitudinal study with 220 samples that there is a significant correlation between organizational support and commitment to work and health status (Panaccio & Vandenberghe, 2009). Furthermore, Eisenberger, Armeli, Rexwinkel, Lynch, and Rhoades (2001) stated in their investigation with 413 samples that the organizational support is effective in welfare, commitment, organizational spontaneity, and the performance of the employees through an interactive process (Eisenberger et al., 2001). In their study, Mozaffari et al. showed that organizational support and fairness in rewarding employees are effective in the organization's revenues as well as satisfaction and social well-being of the personnel (Mozaffari et al., 2014).

Another category discussed in this paper was external support consisting of two subcategories: society's support and media's support. The former involves the support accompanied by public respect. Fortunately, this trend seems promising nowadays because of extended knowledge and understanding about nursing. Moreover, the participants frequently mentioned the positive impact of society's support. Consistent with such a finding, Montes and Augusto (2007) argued that there is a positive correlation between the quality and quantity of the social and emotional support of the nurses and their health status. Besides, results of this study indicated that the feeling of being valued and respected by the society will make the nurses hopeful about their professional future. A study by Cohen (2004) showed that social support and social integration is an important factor in healthy individuals (Cohen, 2004).

Yarcheski, Mahon, and Yarcheski (2001) showed that there is a significantly positive correlation between social well-being, general health, and hopefulness (Yarcheski et al., 2001; Yarcheski, Scoloveno, & Mahon, 1994). These reports suggested that public support of nurses in the society can help maintain their health and hope. In their study titled “Work and Non-work Social Support and Intent to Stay at Work among Jordanian Hospital Nurses,” AbuAlRub (2010) showed that social support is highly crucial among the professional nurses working at hospitals in Jordan. This confirmed that social support is vital for the nurses as the community needs to pay more attention to the well-being of nurses (AbuAlRub, 2010).

The findings indicated that media's support could bring about respect and visibility of nurses in the society by increasing the people's knowledge and understandings. This could be achieved by informing and portraying realistic and scientific images of nursing at the societal level through attractive movies and TV series. An investigation conducted by Sarriera, Abs, Casas, and Bedin (2012) also indicated a significant relationship between showing the real image of individuals by the media, perceived social support, and their health (Sarriera et al., 2012). The results of another study by Rezaei-Adaryani, Salsali, and Mohammadi (2012) suggested that the media is one of the effective factors in providing appropriate images of nursing in the society (Rezaei-Adaryani et al., 2012).

Considering the nature of their work and close relationship with customers, nurses strive to highlight their role and secure their health through evoking the society to see them using communicative power of the media and the internet. This is consistent with the results of Haythornthwaite's investigation (Haythornthwaite, 2005). That is why the nurses have taken advantage of the media's influence to increase their nursing prestige to an extent during recent decades. Moreover, Andres considered the media communication as an effective factor contributing to greater quality of social engagement and individual interactions (Andres, 2002).

## Conclusions

In general, the experience of nurses shows that they are facing several challenges regarding social well-being, the most important of which involves the thirst for support. This has encompassed the nursing community like an umbrella, promoting their social well-being. Perception of such support results in improvement of social well-being among the nurses. However, it is necessary to conduct further studies to gain a better understanding. What strategies are adopted by the nurses to establish their social well-being? How do the media act in developing social well-being for the nurses? How can we manage the culture and attitude of the society to increase the social well-being of nurses?
